# The effects of four-week supplementation of Lion’s mane and a Lion’s mane and Reishi blend on prioritised mood and wellbeing measures in young women

**DOI:** 10.3389/fnut.2026.1887408

**Published:** 2026-10-20

**Authors:** Ellen F. Smith, Sarah Docherty

**Affiliations:** 1 Brain, Performance and Nutrition Research Centre, School of Psychology, Northumbria University, Newcastle upon Tyne, United Kingdom; 2 School of Psychology, Northumbria University, Newcastle upon Tyne, United Kingdom

**Keywords:** Lion’s mane mushroom, mood, participatory effects, Reishi mushroom, wellbeing, women’s health

## Abstract

**Background:**

Functional mushrooms, particularly Lion’s mane (*Hericium erinaceus*) and Reishi (*Ganoderma lucidum*), are increasingly marketed for cognitive and mood-related benefits, despite limited evidence to date.

**Objectives:**

This trial examined the effects of four-week supplementation of Lion’s mane and a Lion’s mane and Reishi mushroom blend on mood and wellbeing in Gen Z women experiencing subjective stress and/or anxiety.

**Methods:**

A preliminary sort-and-rank study was conducted to identify priority outcomes for the population. Following this, a randomised, double-blind, placebo-controlled, parallel-groups trial was conducted. Eighty-four young women (aged 18–26) received either 1.8 g powdered Lion’s mane, 1.8 g powdered Lion’s mane and Reishi mushroom blend, or placebo in capsulated form for 28-days. Mood and wellbeing were assessed at baseline, weekly during the intervention period and at Day 42 (follow-up). Qualitative data regarding subjective changes to wellbeing were collected on Days 28 and 42.

**Results:**

Analysis indicated that the mushroom blend resulted in lower anxiety (GAD-7) than Lion’s Mane, overall (*p* = 0.018) and at Day 28 (*p* = 0.003). Following FDR correction, secondary interactions for anxiety (HADS) and stress (PSS) were attenuated to trend-level, exploratory *post hoc* tests suggested a pattern of benefits of the mushroom blend over Lion’s mane and placebo at various timepoints (*p* = 0.001–0.043), though these should be interpreted cautiously rather than confirmatory. Qualitative data supported this pattern. Significant improvements across all psychological measures were observed regardless of treatment allocation, suggesting participatory benefits alongside intervention effects.

**Conclusion:**

Results indicated that 28-day supplementation with the mushroom-blend improved the primary anxiety outcome (GAD-7), relative to Lion’s mane alone. Trend-level findings on secondary anxiety (HADS) and stress (PSS) measures point to a similar pattern of benefit over both comparators, but require confirmation in adequately powered trials. This trial contributes to a growing field investigating functional mushrooms, however, to further understand efficacy effects, substantial work is required to determine key methodological questions including dosage, intervention duration and blend composition.

**Clinical trial registration:**

ClinicalTrials.gov, identifier NCT06406946.

## 1 Introduction

The prevalence of mental health conditions in young people has increased significantly over the past three decades, where evidence from the Global Burden of Disease indicates that the burden is higher in females and anxiety is highest in those aged 20–24 (1). Those in this age range are often referred to as Generation Z (Gen Z, individuals born between 1997 and 2013). Gen Z women report disproportionately high rates of stress, anxiety and other mental health disorders compared with other generational cohorts (2, 3) and often face barriers accessing psychological support (4–6). This generational cohort show preference for holistic health approaches and there is growing consumer interest in non-pharmacological approaches, including supplements to improve wellbeing (46).

Functional mushrooms are increasingly marketed for cognitive and mood benefits, with the global functional mushroom market projected to reach $62.18 billion by 2032 (7). Of particular research and consumer interest, Lion’s mane *(Hericium erinaceus)* which has a long history of use in Chinese medicine (8) and contains numerous bioactive compounds, including polysaccharides, hericenones and erinacines (9, 10). Preclinical work suggests benefits for nerve growth factor synthesis and secretion, neuroinflammation, up-regulation of pro-brain-derived neurotrophic factor (BDNF) and neuroprotection (11–17). While some human trials have included BDNF as a biological measure, results have been inconsistent (18–20), and the mechanistic pathways underlying any cognitive or wellbeing effects of Lion’s Mane are yet to be established. Limited empirical evidence also exists to support the claims of benefits to cognitive function and wellbeing.

Until recently, human trials have focused on older adults or clinical populations, where modest cognitive improvements have been observed (19, 21–23). Emerging trials in younger, healthy populations remain limited with inconsistent findings. Acute supplementation has shown some limited cognitive benefits (24–26), while chronic supplementation has resulted in no cognitive benefits (24, 27).

Evidence for mood effects is similarly inconsistent. Chronic supplementation has resulted in improvements on depression (20, 22) and anxiety (20) outcomes in older adults, and a trend toward reduced subjective stress in younger adults (24). However, acute supplementation shows mixed (25) or no (26) mood effects. Notably, several trials have not assessed mood outcomes (18, 19, 21, 23). These discrepancies likely reflect substantial methodological differences across studies, including variation in dosage, formulation (e.g., powdered mushroom or extract) and assessment timings.

In parallel with single-ingredient supplements, mushroom blend products combining Lion’s mane with other fungi or non-fungal nootropics are widely available. Reishi (*Ganoderma lucidum*), commonly co-formulated with Lion’s mane, has demonstrated immunomodulatory properties (28). However, evidence for cognitive modulation is limited and inconclusive, with enhancements observed in animal models (29), but not in human research (30, 31). Despite this, anecdotal reports and marketing claims suggest mood and fatigue benefits, but these remain under researched in the literature, where to the best of the authors’ knowledge, only a single randomised controlled trial has directly examined the psychological effects of a mushroom blend product. With reductions in anxiety, depression and fatigue scores observed following 6- and 12- week supplementation with a Cordyceps, Reishi, Shiitake, Maitake and Lion’s Mane blend (32).

The present study therefore examined the effects of Lion’s mane and a Lion’s mane and Reishi blend on mood and wellbeing in Gen Z women experiencing self-reported stress and/or anxiety. To ensure relevance to this population, we first conducted a sort-and-rank trial in which participants identified the symptoms of poor mental health that were most important to them. This formative phase of the trial guided the selection of outcome variables for the intervention phase. By combining a sort-and-rank approach with a randomised controlled trial including qualitative data, this study aimed to provide both participant-informed outcomes and evaluation of the efficacy of mushroom-based interventions in modulating mood in this population.

## 2 Methods

### 2.1 Trial design

#### 2.1.1 Sort and rank trial

Prior to the intervention, a sort and rank trial consisting of 38 Gen Z women who self-reported as feeling stressed and/or anxious was conducted to determine which areas of poor mental well-being were most important to the trial population. This was conducted in a separate cohort to the intervention trial, but inclusion criteria remained the same to ensure the same population was being studied. Ethical approval was obtained from the Northumbria University Ethics Online System (Reference 5,917), Fifty-four statements were presented to participants based on DSM-5 diagnostic criteria for acute stress disorder, generalised anxiety disorder, depressive disorders and ADHD, due to the high prevalence and co-morbidity of these conditions within the target population. Participants initially sorted the statements into three broad categories: “Most important,” “No strong feelings” and “Least important.” They then completed a structured ranking task, placing each statement on a forced quasi-normal distribution grid ranging from −6 (least important) to +6 (most important). Data from the sort and rank task were analysed descriptively. Mean rank scores were calculated to examine overall trends in perceived importance. Cumulative percentages were calculated to further interpret the relative importance of each statement. The top-rated symptoms can be viewed in Table 1. Full reporting of methodology and results is included in Supplementary materials.

**Table 1:** Top-ranked symptoms of poor mental well-being in Gen Z women.

Rank	Statement	% Scored > + 2	Mean rank
1	Excessive anxiety and worry	59.5	2.89
2	Feeling mentally tired/exhausted	56.8	2.64
3	Feeling physically tired/exhausted	51.4	1.67
4	Feeling close to panic	43.2	1.27
5	Overwhelming feelings of stress	43.2	2.45
6	Feelings of worthlessness	43.2	2.08
7	Low self esteem	43.2	1.91
8	Lack of motivation	40.5	1.94
9	Difficulty working up the initiative/motivation to do things	40.5	2.00
10	Unable to muster any energy	35.1	1.16

“% scored > + 2” indicates the percentage of participants who assigned the statement a rank of greater than +2 on the −6 to +6 scale (i.e., who placed the item in their top 16 most important statements). Higher percentages reflect higher perceived importance.

#### 2.1.2 Intervention

The trial employed a randomised, placebo-controlled, double-blind, parallel-groups design with participants randomised to one of three treatment conditions according to a randomisation schedule. Data collection occurred between April 2024 and June 2025 at the Brain, Performance and Nutrition Research Centre (BPNRC), within Northumbria University. Ethical approval was obtained from the Northumbria University Ethics Online System (Reference 6,969), with adherence to the principles outlined in the Declaration of Helsinki (1975).

Given the limited existing literature, sample size calculations referenced the two most appropriate studies. Using G* Power, calculations were conducted with reference to our pilot trial’s large effect size of stress reduction following 28-days supplementation (*f* = 0.35 (24)) and the more conservative effect size reported by La Monica, Raub et al. (25) for improved happiness following an acute dose (*f* = 0.22). A sample size of 54 participants (18 per group) and 129 participants (43 per group), respectively, would achieve 80% power at *p* < 0.05. Given this disparity, the trial aimed to recruit between 18 and 45 participants per group, targeting 135 participants to account for attrition.

Due to recruitment challenges, 84 participants were randomised into the trial. A post-hoc power analysis based on the observed effect size for the primary outcome GAD-7 Treatment x Day interaction (*η*^2^p = 0.06, *f* = 0.26) indicated 99% power at *α* = 0.05 with *N* = 84. However, given the well-documented limitations of post-hoc power calculations, this should be interpreted cautiously.

### 2.2 Participants

A sample of 84 Gen Z women (born between 1997 and 2006, with a mean age of 21.93 years) were recruited and randomised into the trial. Participants self-reported as experiencing feelings of anxiety or stress, without a diagnosis of a psychiatric condition, indicated that they were in good health and were native speakers/fluent in English. Participants were without relevant chronic disease, did not take prescription medication or dietary supplements (aside from explicit exceptions), and did not meet any further exclusion criteria. Full information regarding the inclusion and exclusion criteria is detailed within Supplementary materials. Detailed participant characteristics at the point of enrolment are available within Supplementary materials. An overview of participant enrolment and flow can be viewed within the CONSORT diagram in Figure 1.

**Figure 1:**
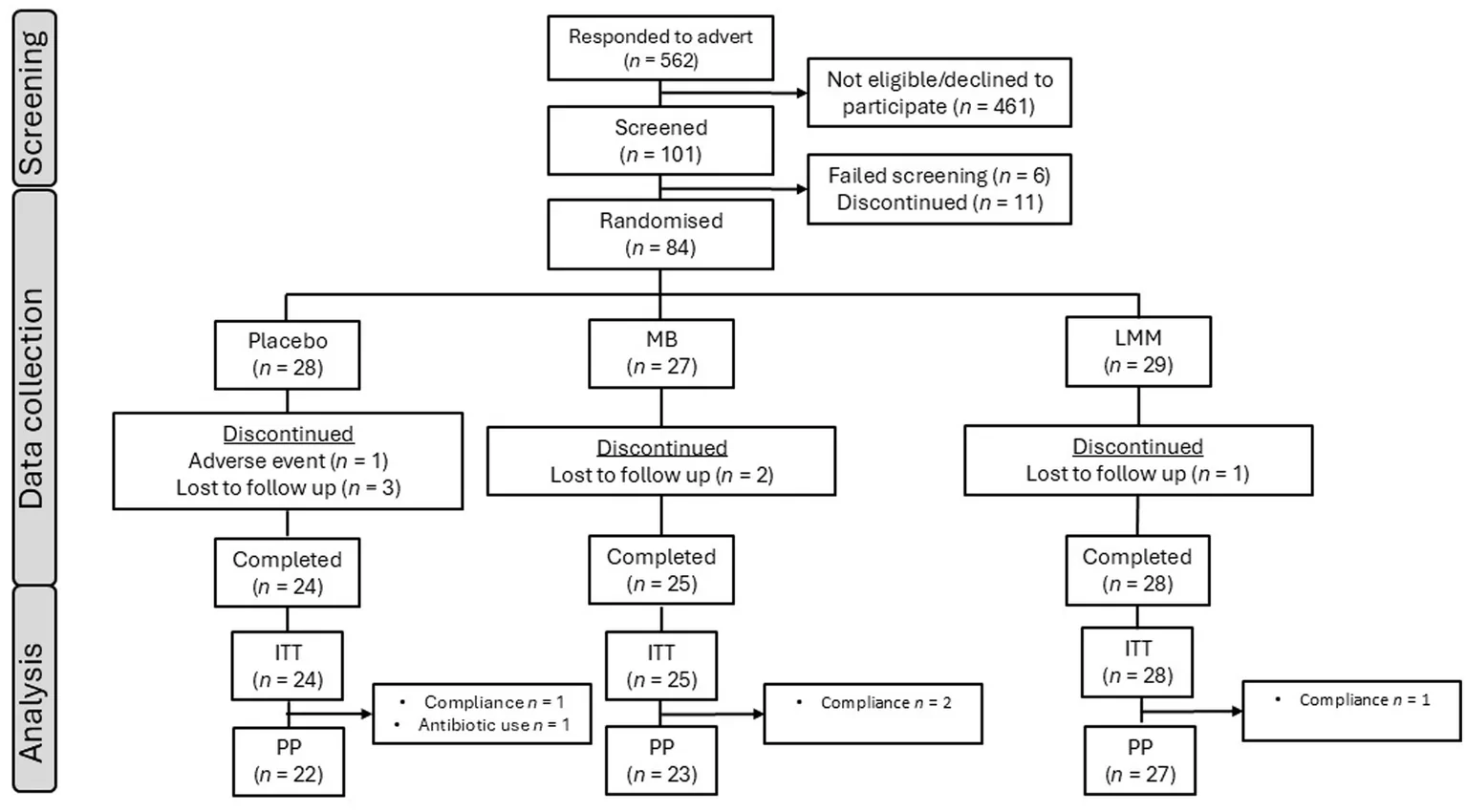
CONSORT diagram. ITT, Intent-to-Treat; PP, Per Protocol; MB, mushroom blend; LMM, Lion’s mane mushroom.

### 2.3 Treatments

The treatment regimen consisted of taking three capsules daily, containing either placebo (microcrystalline cellulose), 600 mg Lion’s mane mushroom (SO-DSX1®, Sempera Organics Inc., Morgan Hill, CA, United States), or 600 mg Lion’s mane and Reishi mushroom blend (SO-DSX2®, Sempera Organics Inc., Morgan Hill, CA, United States) with the active conditions equating to a daily dose of 1.8 g. Lion’s mane capsules contained a proprietary blend of organic *Hericium erinaceus* mushroom complex; and the mushroom blend capsules contained a blend of 60% organic *Hericium erinaceus* and 40% *Ganoderma lucidum* mushroom complex, standardized to 20% β-glucans. Treatments were matched for appearance, with all capsulated in dark green vegetarian capsules (size 00). Participants were instructed to consume capsules in the morning, with breakfast.

The randomisation list was computer generated using the online website https://www.randomizer.org. The mushrooms products were provided in bulk and the research team capsulated these. Treatments were labelled by the Director of the research centre, who was also responsible for blinding and creating the emergency code break envelopes. They had no further involvement in the study. All participants and the research team were kept blinded during the entire study period. No emergency unblinding was needed at any time during the study. The blinded treatment code was only broken after completion of the statistical analyses. Participants were sequentially assigned to a treatment condition according to the randomisation list at the Day 1 testing visit.

### 2.4 Mood outcomes

Mood outcomes were determined with reference to results of the Sort and Rank trial, as detailed within Section 2.1 and the Supplementary materials.

The primary outcome was ratings of anxiety via the Generalised Anxiety Disorder Assessment (GAD-7). The secondary outcome measures were assessed using the anxiety subscale of the Hospital Anxiety and Depression Scale (HADS), Perceived Stress Scale (PSS), Stress Visual Analogue Scales (S-VAS), Chalder Fatigue Scale and the Rosenberg’s Self Esteem Scale. Descriptions of the mood questionnaires are available in the Supplementary materials.

### 2.5 Procedure

Participants attended three appointments: a remote screening appointment, and two testing visits, Figure 2 illustrates the study protocol overview. The remote screening session was conducted via telephone and included collecting informed consent and eligibility checks. Participants had no dietary restrictions prior to the testing appointments, with the aim of this being more reflective of their everyday routine. Physiological measures (blood pressure, height, weight and waist-to-hip ratio) were collected, followed by completion of the full well-being assessment (GAD-7, HADS, Chalder Fatigue Scale, Rosenberg’s Self-Esteem Scale, PSS and Stress-VAS). Participants were randomised and given their treatment to consume at home for the 28-day supplementation period. Participants completed at home, interim mood questionnaires on Days 7, 14 and 21, which consisted of completion of the HADS and S-VAS. They returned to the research centre on Day 28 and completed their final well-being assessment in person. Participants returned their remaining capsules and diary so that compliance could be calculated. At the end of the session, they completed a treatment guess form and provided qualitative feedback in response to three questions “What is your reason(s) for thinking this [treatment option selected]?”, “Have you noticed any change in your thinking and memory whilst taking the product?” “Have you noticed any change in your mood or wellbeing whilst taking the product?”. Each testing visit was approximately 30 min in duration and participants received £30 compensation upon successful completion of the study. On Day 42, 14 days after treatment cessation, participants completed a final at-home follow-up assessment, including completion of the GAD-7, HADS, S-VAS and completion of two further open-ended questions “Have you noticed any changes in your thinking and memory after stopping taking the product?” and “Have you noticed any change in your mood or wellbeing after stopping taking the product?”. This exploratory timepoint was included to assess potential changes in wellbeing following treatment cessation.

**Figure 2:**
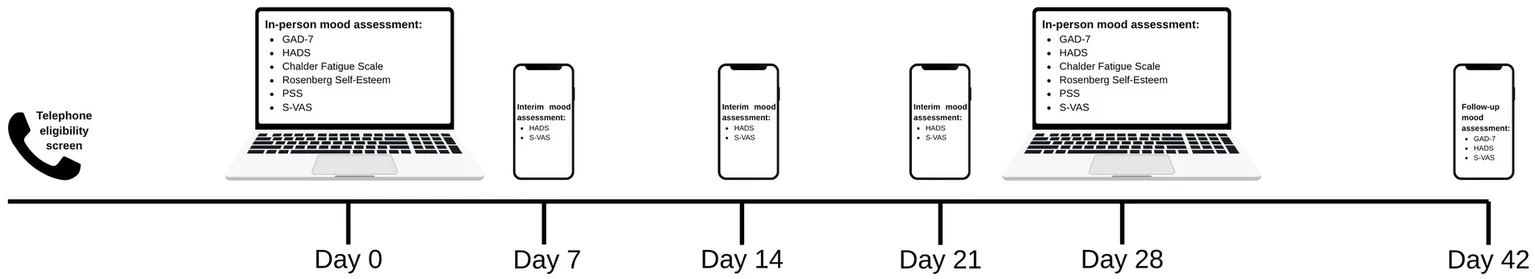
Overview of the intervention trial protocol.

### 2.6 Statistical analysis

Prior to main analysis, baseline differences were assessed using one-way ANOVAs, which indicated significant differences between treatment groups for fruit and vegetable consumption (*p* = 0.021), waist-to-hip ratio (*p* = 0.033) and stress VAS score (*p* = 0.026), full details of all outcomes and *post hoc* comparisons are included within Supplementary materials. Table 2 provides an overview of baseline characteristics and one-way ANOVA results between treatment conditions.

**Table 2:** Summary of baseline differences between treatment conditions in ITT population.

Demographic variable	Treatment group	*N*	Mean	SD	F	*p*
Age (years)	Placebo	28	22.21	2.68	0.76	0.46
	MB	27	21.44	2.25		
	LMM	29	22.10	2.51		
Years in Education (years)	Placebo	28	16.25	1.89	0.79	0.45
	MB	27	16.53	2.27		
	LMM	29	15.93	2.05		
CCQ Score (mg)	Placebo	28	101.87	99.62	0.08	0.92
	MB	27	111.92	77.82		
	LMM	29	107.01	99.69		
**Fruit and Veg portions** (75 g portion)	**Placebo**	**28**	**3.69**	**1.79**	**4.04**	**0.02**
	**MB**	**27**	**2.63**	**1.13**		
	**LMM**	**29**	**3.31**	**1.19**		
Systolic BP (mmHg)	Placebo	28	113.26	12.43	0.11	0.88
	MB	27	111.98	7.35		
	LMM	29	112.70	8.98		
Diastolic BP (mmHg)	Placebo	28	73.66	10.19	1.34	0.26
	MB	27	76.92	6.56		
	LMM	29	74.01	7.06		
Heart rate (BPM)	Placebo	28	74.92	12.23	0.69	0.50
	MB	27	78.59	11.72		
	LMM	29	76.08	11.46		
BMI (kg/m^2^)	Placebo	28	22.41	4.27	0.93	0.39
	MB	27	23.42	3.86		
	LMM	29	24.19	6.26		
**WHR (waist/hip ratio)**	**Placebo**	**28**	**0.78**	**0.05**	**3.54**	**0.03**
	**MB**	**27**	**0.81**	**0.05**		
	**LMM**	**29**	**0.77**	**0.05**		
GAD-7 Score	Placebo	28	9.18	2.76	1.36	0.26
	MB	27	10.59	4.13		
	LMM	29	10.38	3.31		
HADS Score	Placebo	28	10.46	2.79	1.71	0.18
	MB	27	11.81	2.84		
	LMM	29	11.48	2.86		
Chalder Score	Placebo	28	16.93	3.98	0.41	0.65
	MB	27	18.07	5.63		
	LMM	29	17.38	4.26		
Self Esteem Score	Placebo	28	16.04	3.50	0.33	0.71
	MB	27	15.19	4.29		
	LMM	29	15.45	3.96		
PSS Score	Placebo	28	22.57	4.70	2.28	0.10
	MB	27	25.11	4.69		
	LMM	29	24.86	5.24		
**Stress VAS Score**	**Placebo**	**28**	**51.31**	**15.34**	**3.81**	**0.02**
	**MB**	**27**	**60.91**	**17.48**		
	**LMM**	**29**	**62.20**	**15.63**		

Baseline variables with significant differences between treatment groups are highlighted in bold.

Each mood questionnaire outcome measure was analysed using the MIXED procedure in SPSS (Version 29). The model included Treatment (Placebo, Lions Mane, Mushroom blend) and Day as fixed effects, as well as their interaction (Treatment x Day). Baseline scores for the relevant outcome measure were entered as a covariate, as well as fruit and vegetable intake, waist-to-hip ratio (WHR) and Stress VAS baseline scores. Estimated marginal means were compared using Sidak-adjusted pairwise tests.

To account for multiple comparisons across the secondary outcomes, a false discovery rate (FDR) correction (Benjamini-Hochberg procedure) was applied. Secondary outcomes were grouped into a psychological negative affect domain, which comprised anxiety (HADS), stress (PSS and SVAS), and fatigue (Chalder Fatigue Scale) [*n* = 4 tests per effect]. Self-Esteem (Rosenberg’s Self-Esteem Scale) was analysed as a single-outcome family given the conceptual distinction from the distress-symptom measures. Benjamini-Hochberg correction was applied separately within each family for the treatment, visit and treatment*visit effects. Both raw *p*-values and FDR-adjusted q-values are reported for negative affect domain secondary outcomes effects to allow readers to interpret findings both at the nominal level and after correction for multiplicity. *Post hoc* pairwise comparisons were conducted for effects significant at the nominal level (raw *p* < 0.05); interpretation of these findings detail whether they remained robust after FDR correction.

The primary population of interest was the Intent-to-Treat (ITT) population. Results from the Per-Protocol (PP) population, can be viewed within Supplementary materials.

For qualitative data, responses were independently coded by two researchers, then compared and agreed on. Any disagreements were resolved between the two reviewers. Codes were collapsed into key themes and binary coding (theme present/absent) was used for frequency analyses. Fisher’s exact tests were conducted in RStudio (Version 4.5.1) to compare theme frequency by treatment group with holm-adjusted post-hoc comparisons.

To assess adequate blinding, a chi-squared test was run to assess whether treatment administered was associated with treatment guess.

### 2.7 Compliance and blinding

Compliance was assessed via a count of returned capsules; this was assessed to be adequate with an average compliance of 96.7%.

Adequate blinding was confirmed via analysis of treatment guess responses, where a chi-squared test confirmed that participants were not able to correctly identify their treatment group *χ*^2^ (6, *N* = 77) = 6.72, *p* = 0.34. A confusion matrix heatmap illustrated how participants in each treatment group guessed is shown in Figure 3. It was hypothesised that participants may be able to identify if they were in an active condition but not be able to correctly identify which of the active treatment groups they were assigned to (Lion’s Mane or Mushroom Blend). To assess this, Lion’s Mane and Mushroom Blend treatment groups and guesses were recoded to ‘Active’. Chi-squared analysis indicated that participants were not able to correctly identify if they had received Active or Placebo *χ*^2^ (1, *N* = 77) = 0.13, *p* = 0.72.

**Figure 3:**
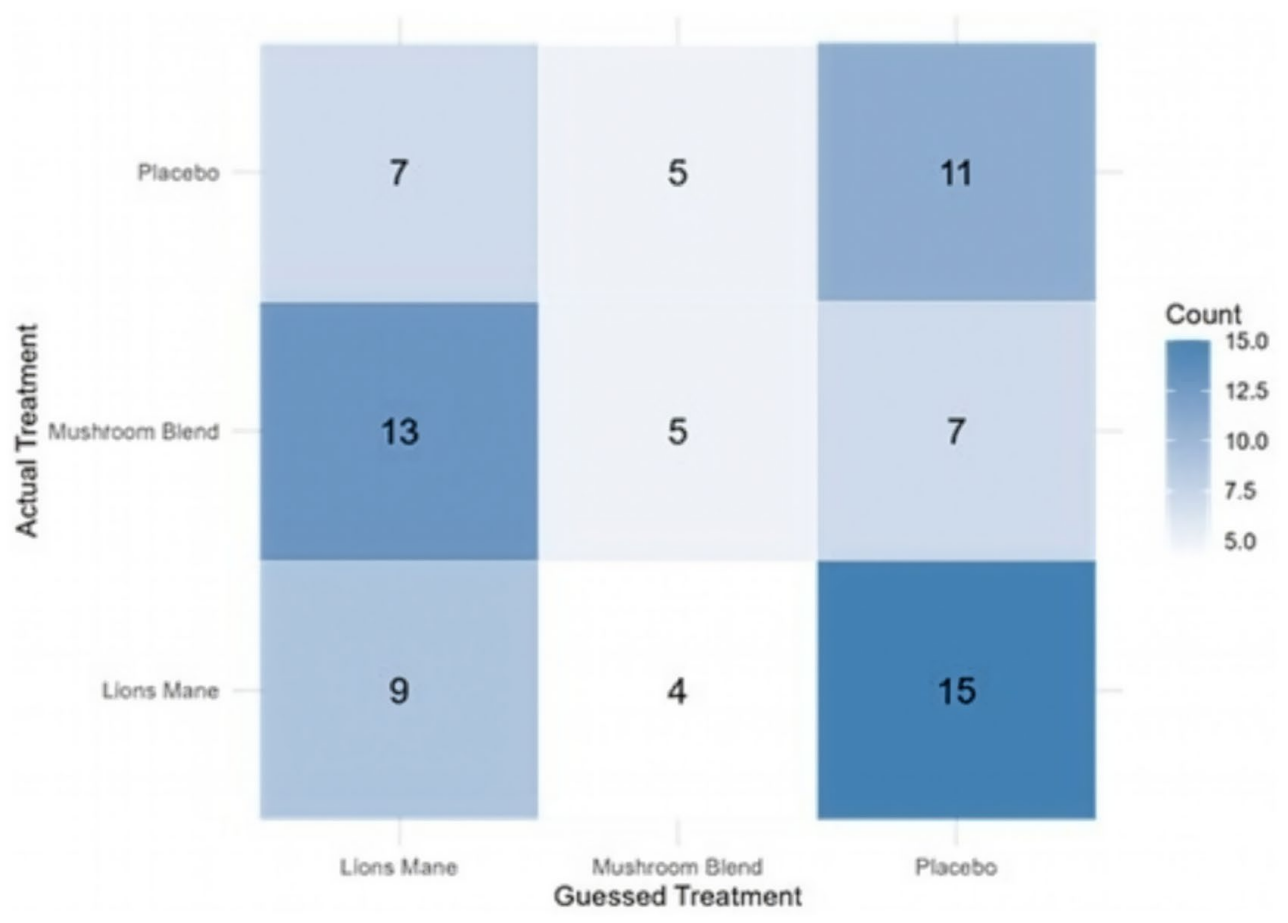
Confusion matrix heatmap of treatment guess.

### 2.8 Safety analysis

In total, 50 adverse events (reported by 31 individual participants) were reported during the supplementation period. One participant discontinued the trial due to an adverse event (headache); this participant was in the placebo condition.

No statistically significant differences were observed in the frequency of any reported adverse events between treatment groups. Fisher’s exact tests were conducted of each adverse event using simulated *p*-values (10,000 permutations), and all *p*-values remained non-significant after false discovery rate (FDR) correction. Results summary table can be viewed within Supplementary materials. This suggests that the mushroom-based supplements were generally well tolerated and not associated with increased risk of side effects relative to placebo in this sample.

## 3 Results

Only the outcomes which revealed significant main or interaction effects are reported below. Results of all statistical analyses can be found in the Supplementary materials.

### 3.1 Response to intervention

For the primary outcome measure, the GAD-7 a main effect of Treatment *F* (2, 97.92) = 3.98, *p* = 0.022, *η*^2^p = 0.07, 90% CI [0.01,0.15] was observed. Post-hoc tests indicated that irrespective of Day, those in the Mushroom Blend condition scored lower (mean = 6.49) compared to the Lion’s Mane condition (mean = 7.76) [*p* = 0.018, *d* = 0.51, 95% CI (0.13, 2.37)]. A significant Treatment x Day interaction effect was also observed, *F* (4, 153.74) = 2.68, *p* = 0.034, η^2^p = 0.06, 90% CI [0.00,0.12]. Post-hoc tests indicated that on Day 28 those in the Mushroom Blend condition scored lower (mean = 4.16) compared to those in the Lions Mane condition (mean = 6.50) [*p* = 0.003, *d* = 0.94, 95% CI (0.65, 4.02)].

Analysis of the secondary outcomes revealed a significant interaction effect of Treatment x Day *F* (10, 309.11) = 2.12, *p* = 0.023, η^2^p = 0.06, 90% CI [0.00,0.08] on the anxiety subscale of the HADS. Which did not remain significant following FDR correction for multiple comparisons across secondary outcomes (q = 0.072). Post-hoc tests indicated that on Day 28, those in the Mushroom Blend condition scored lower (mean = 6.04) compared to those in the Lion’s Mane condition (mean = 8.10) [*p* = 0.010, *d* = 0.82, 95% CI (0.37, 3.76)]. Additionally, on Day 42 those in the Mushroom Blend condition scored lower (mean = 6.18) than those in the Placebo condition (mean = 9.07) [*p* = 0.001, *d* = 1.16, 95% CI (0.93, 4.87)].

A significant interaction effect of Treatment x Day was observed on the PSS, *F* (2, 82.63) = 3.45, *p = *0.036, η^2^p = 0.07, 90% CI [0.00,0.17]. Which did not remain significant following FDR correction for multiple comparisons across secondary outcomes (*q* = 0.072). Post-hoc tests indicated that on Day 28 those in the Mushroom Blend condition scored lower (mean = 16.86) compared to those in the Placebo condition (mean = 19.79) [*p* = 0.017, *d =* 0.83, 95% CI (0.41, 5.45)] and those in the Lion’s Mane condition (mean = 19.35) [*p* = 0.043, *d* = 0.70, 95% CI (0.06, 4.92)].

Figure 4 illustrates response to treatment for mood outcomes with significant main or interaction effects of treatment.

**Figure 4:**
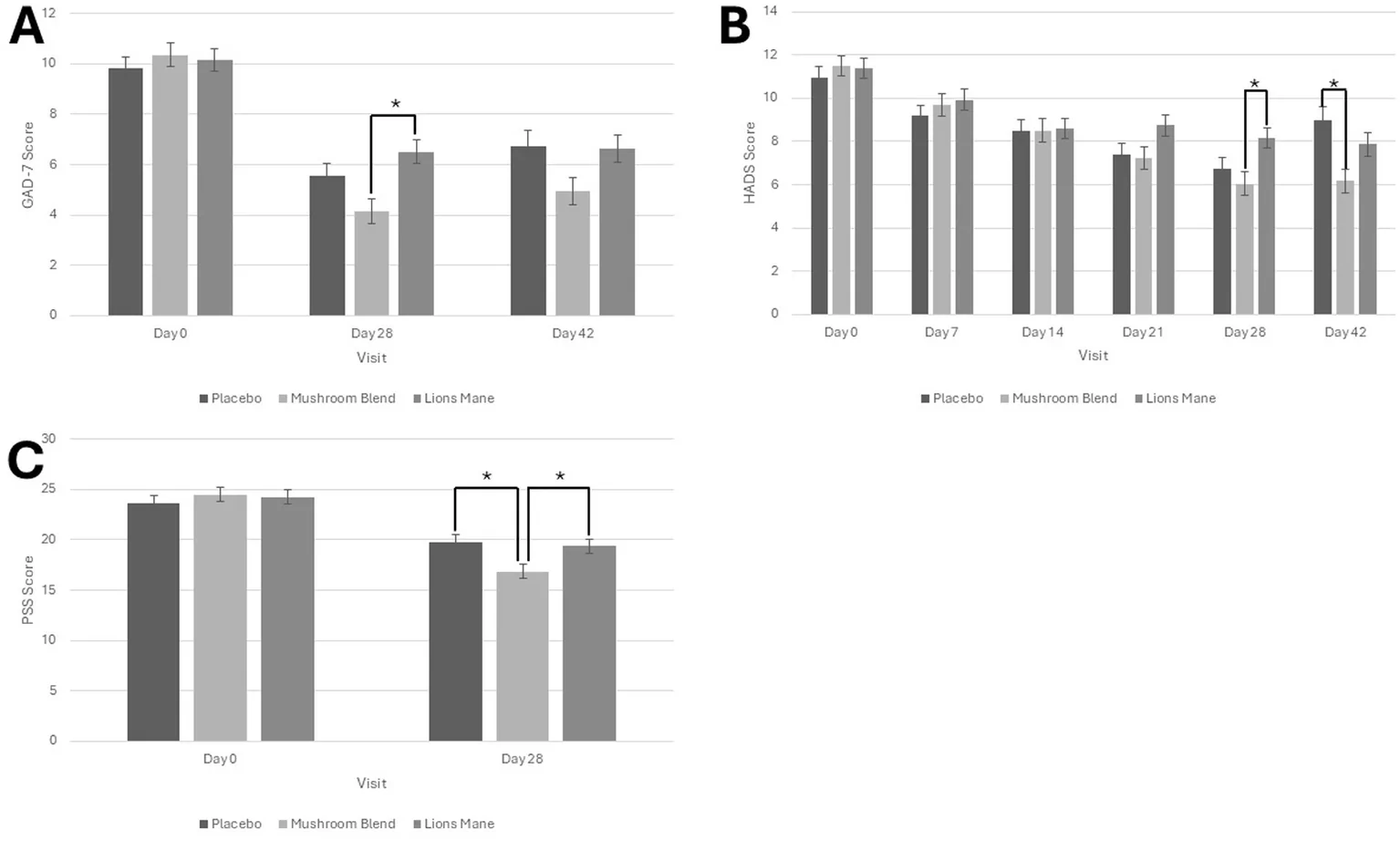
Response to intervention effects. **(A)** GAD-7, **(B)** HADS, **(C)** PSS. *p < 0.05.

### 3.2 Effects of time

A significant main effect Day was observed for anxiety as measured by the GAD-7, *F* (2, 153.90) = 84.96, *p* < 0.001, η^2^p = 0.52, 90% CI [0.43,0.59]. Post-hoc comparisons showed that compared to Day 0 (mean = 10.08), participants scored lower on Day 28 (mean = 5.42) and Day 42 (mean = 6.12), irrespective of treatment group (all *p* < 0.001).

Similar time effects were observed across secondary outcome measures. Anxiety scores as measured by the HADS decreased significantly from Day 0 (mean = 11.29), at all subsequent time-points, with a significant main effect of Day *F* (5, 309.11) = 26.28, *p* < 0.001, η^2^p = 0.29, 90% CI [0.22,0.35], q < 0.001 and post-hoc comparisons all significant at *p* < 0.001. Additional comparisons between other timepoints are included within Figure 5. Ratings of stress using the PSS also declined over time, *F* (1, 82.67) = 85.32, *p* < 0.001, η^2^p = 0.50, 90% CI [0.20,0.66], q < 0.001, with significantly lower ratings on Day 28 (mean = 18.67), compared with Day 0 (mean = 24.14). Scores on the Stress VAS also followed the same pattern, with a main effect of Day *F* (5, 306.54) = 18.65, *p* < 0.001, η^2^p = 0.23, 90% CI [0.16,0.29], q < 0.001, and *post hoc* comparisons indicating significantly lower scores at all timepoints compared to Day 0 (all *p* < 0.001); with comparisons between other timepoints included within Figure 5. Ratings of fatigue, measured using the Chalder Fatigue Scale, decreased from Day 0 (mean = 17.48) to Day 28 (mean = 10.74), *F* (1, 82.53) = 141.10, *p* < 0.001, η^2^p = 0.63, 90% CI [0.52,0.70], q < 0.001. Whilst self-esteem scores increased from Day 0 (mean = 15.59) to Day 28 (mean = 18.56), *F* (1, 82.80) = 68.46, *p* < 0.001, η^2^p = 0.45, 90% CI [0.32,0.55], q < 0.001.

**Figure 5:**
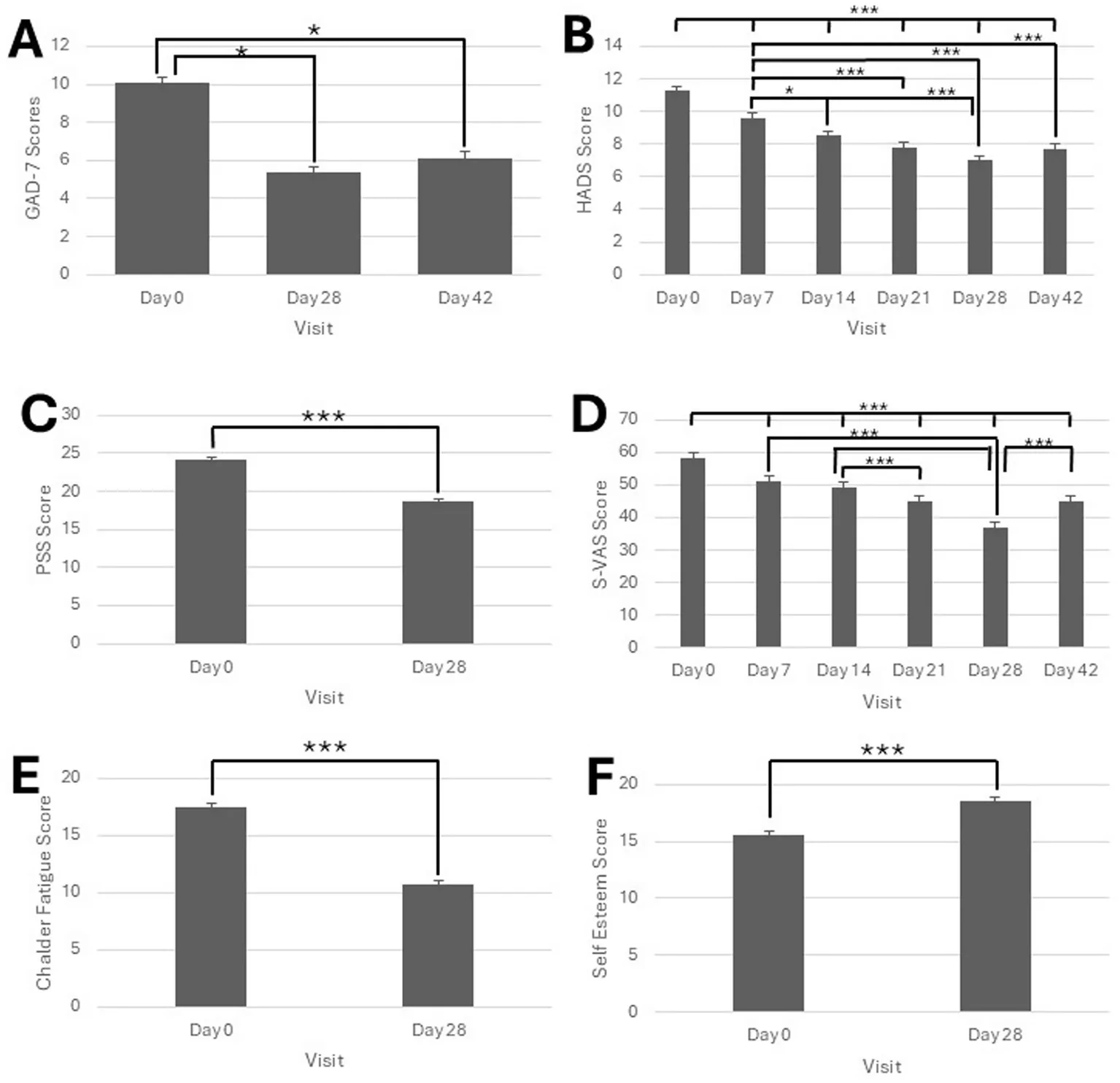
Effects of time. **(A)** GAD-7, **(B)** HADS, **(C)** PSS, **(D)** S-VAS, **(E)** Chalder’s Fatigue Scale, **(F)** Self Esteem Scale. * *p* < 0.05, *** *p* < 0.001.

Figure 5 illustrates effects of time for all mood outcomes across the course of the trial.

### 3.3 Analysis of qualitative responses – perceived effects of treatment

Analysis by treatment group indicated a significant association between treatment groups for the theme of ‘No Change’ in the ‘reason for thinking this’ question on Day 28. *Post hoc* comparisons indicated that participants in the Lion’s Mane condition reported ‘No change’ more frequently than those in the Mushroom Blend condition [*p* = 0.025, OR = 0.24, 95% CI (0.06, 0.88)], with a trend to more than those in the Placebo condition [*p* = 0.054, OR = 0.32, 95% CI (0.08, 1.12)]. The same effect was observed as a trend in response to the mood and wellbeing question (*p* = 0.054), with those in the Lion’s mane condition reported ‘No change’ more frequently than those in the Mushroom Blend condition [*p* = 0.022, OR = 0.23, 95% CI (0.04, 0.92)]. A further trend was observed for the ‘Improvement’ theme on the mood and wellbeing question (*p = 0*.059), where those in the Mushroom Blend condition reported mood improvements more frequently than those in the Lion’s mane condition [*p* = 0.027, OR = 3.86, 95% CI (1.09, 14.98)].

## 4 Discussion

The aim of this trial was to expand on the limited research assessing the potential mood enhancing effects of Lion’s Mane mushroom and a Lion’s Mane and Reishi blend in Gen Z women who feel stressed and/or anxious, over a 28 day intervention period. The main efficacy analysis indicated that 28-day consumption of the mushroom blend resulted in improvements on the primary outcome of anxiety (GAD-7). For secondary outcomes, following FDR correction for multiple comparisons, the treatment*visit interactions for anxiety (HADS) and stress (PSS), were attenuated from significance to non-significant, trending effects after adjustment. While *post hoc* comparisons suggested beneficial effects of mushroom blend for anxiety (HADS) and stress (PSS), when compared with the Lion’s mane alone. And lower subjective stress (Day 28, PSS) and anxiety (Day 42, HADS) compared with placebo. These findings should be interpreted with caution given they did not survive correction for the number of secondary outcomes tested. Qualitative analysis supported this pattern, with those in the mushroom blend condition reporting mood improvements more frequently than those who received Lion’s mane alone.

Reductions in anxiety, as measured by GAD-7, were observed following 28-days supplementation with the mushroom blend, when compared with those in the Lion’s Mane only condition. This is consistent with the findings of Hisamuddin, Ramli et al. (32) who reported significantly reduced anxiety scores, following 6 weeks supplementation with a mushroom blend containing five adaptogenic mushrooms (including Lion’s Mane and Reishi). The current trial extends upon this work, as anxiety reductions were observed after just 4 weeks of supplementation, suggesting mood benefits may emerge earlier than previously demonstrated. These effects were also observed with a simplified blend containing only Lion’s Mane and Reishi. However, given that these two trials differ in dosage (500 mg mushroom blend extract per day vs. 1.8 g mushroom blend powder in the current trial) and blend composition, it remains unclear whether the observed mood beneficial effects are underpinned by synergistic interactions between mushrooms or the mechanism of action of a single ingredient. Within the present trial, lower anxiety scores (GAD-7) were observed in the blend condition compared with the Lion’s Mane alone condition, tentatively suggesting that the addition of Reishi could be contributing to this effect. However, it must be acknowledged that the absolute quantities of Lion’s Mane in the two active arms differ. The Lion’s mane condition received 1.8 g of Lion’s mane, whereas the mushroom blend condition received approximately 1.08 g Lion’s mane (60% of the 1.8 g blended product), which makes direct comparisons between the two active arms difficult. Unlike Lion’s Mane, which is characterised primarily by hericenones and erinacines, Reishi is characterised by triterpenoids, including ganoderic acids, and polysaccharides, particularly β-glucans (33). Evidence from preclinical models, suggest Reishi’s triterpenoid constituents have the ability to reduce pro-inflammatory markers including TNF-α, IL-1β and IL-6 (34). Given the potential association between peripheral inflammatory markers and anxiety symptomology (35), one plausible mechanism is that Reishi contributes an anti-inflammatory pathway to anxiety reduction that is independent of, and potentially additive to Lion’s Mane. Notably, previous work has demonstrated reductions in anxiety following 6-weeks supplementation of Lion’s Mane alone (20), raising the possibility that the addition of Reishi may enhance or accelerate this effect, rather than acting independently. However, without the inclusion of a Reishi only intervention arm in this trial or previous work, this is speculative and warrants further investigation, as a priority. Disentangling the individual and potential synergistic interactions of functional mushrooms will require future trials with single-ingredient and dose-matched comparator arms.

Of interest, exploratory post-hoc comparisons, on the secondary outcome HADS, that did not survive FDR correction, suggested lower anxiety scores in the mushroom blend condition relative to placebo at Day 42, 2 weeks following cessation of supplementation. This finding may tentatively suggest some longevity of the effect beyond the active intervention period, but this remains speculative and should be interpretated as such. Future research should investigate this further through Reishi-only and combination mushroom trials, employing regular mood measures, to assess the extent and duration of any anxiolytic effects of individual and combinations of adaptogenic mushrooms.

Alongside the primary anxiety findings, exploratory analyses suggested lower perceived stress (PSS) in the mushroom blend condition, relative to both the placebo and Lion’s Mane conditions. This pattern was significant using uncorrected *p*-values but was attenuated to trend-level following FDR-adjustment, and therefore should be interpreted as exploratory findings, rather than an established treatment effect. This contrasts with the findings of Hisamuddin, Ramli et al. (32), who reported reductions in PSS scores from baseline at 6 and 12 weeks, but did not appear to demonstrate significant between-group differences on this measure relative to placebo. Given that in placebo-controlled trials primary evidence for efficacy should be based on significant differences versus placebo, the current trial is the first to demonstrate this effect. Caution should be taken when results are being presented as improvements within an active treatment group, as this may reflect participatory or repeated testing effects. However, Hisamuddin et al. (32) did observe a significant reduction in serum cortisol levels compared to the placebo group at 6 weeks, indicating an effect on stress biomarkers that was not measured in the present trial. As with the anxiety finding, significant stress reductions were absent in the Lion’s Mane only condition, which is contrary to our previous work which reported a trend toward reduced subjective stress following 28-day Lion’s Mane supplementation in younger adults (24). However, the literature in this area remains limited, particularly when investigating stress reducing effects of Lion’s Mane and other adaptogenic mushrooms, which does not allow firm conclusions to be made. Nevertheless, the present findings raise the possibility of potential stress alleviating effects of a mushroom blend.

It must also be acknowledged that there are several outcomes which showed no significant effect of treatment (Stress VAS, Fatigue and Self Esteem). In contrast, Hisamuddin, Ramli et al. (32) found significant improvements in physical fatigue at both 6- and 12- weeks, again these differences could be attributed to the differences in mushroom blends used. Meta analysis evidence has shown that β-glucans reduce subjective fatigue and improve vigour (36). Whilst Reishi is particularly high in β-glucan content, this is also found in high quantities in Cordyceps, Shiitake and Maitake (37, 38), which were included in Hisamuddin, Ramli et al. (32) blend, but not the current study. Muroya, Nakada et al. (36) also suggested that to effectively reduce feelings of fatigue, β-glucans need to be consumed for at least 4 weeks. Given this, the intervention period may need to exceed 4 weeks to elicit measurable benefits to fatigue, or different mushrooms higher in β-glucans may be required.

Additionally, self-esteem was highlighted by the target population as a priority area and was included for that purpose, despite limited mechanistic evidence to support an effect. It was hypothesised that any benefit to self-esteem could be mediated by improvements in positive mood, given that Lion’s Mane has previously been associated with increased happiness ratings (25). However, the mood battery in the current trial primarily targeted negative affect (anxiety, stress, and fatigue), with self-esteem the only measure relating to positive mood. It is therefore possible that benefits to other aspects of positive mood, such as happiness, occurred but were not measured within this trial. As there were no significant treatment effect on self esteem, potential mediation via positive mood has not been explored.

Importantly, irrespective of treatment allocation, significant improvements were observed across all psychological outcomes, including anxiety (GAD-7, HADS), stress (PSS, Stress VAS), fatigue (Chalder Fatigue Scale) and self-esteem (Rosenberg’s Self Esteem Scale). These widespread improvements suggest that trial participation itself may have contributed to psychological benefits, independent of active treatment, through several potential mechanisms, including expectancy effects, the Hawthorne effect and self-monitoring effects.

Expectancy effects, where participants’ assumptions or prior beliefs about an intervention shape their outcomes, are well documented in pharmacological research, with placebo response rates reaching 35–40% in anti-depressant trials (39). However, these are rarely reported within nutritional intervention trials. Evidence suggests that manipulating expectancy statements and trial descriptions alongside an intervention can alter participant outcomes, including mood scores (40, 41). Several factors may have amplified expectancy effects in this trial. As part of the informed consent process, participants were aware they were enrolled in a trial evaluating mushroom-based supplements, though allocation to the treatment arm remained blind throughout (in line with the double-blind design and as confirmed by Chi-square analysis). Expectancy effects discussed here relate to the general beliefs about the efficacy of mushroom-based products as a whole, which would be expected to occur similarly across all arms (including placebo), rather than reflecting awareness of treatment group allocation. The sample comprised Gen Z women, a demographic highly exposed to social media marketing of mushroom-based products, where evidence suggests pre-existing beliefs about supplement efficacy may shape participants expectations and subsequently influence outcomes, irrespective of the specific treatment received (42). This offers a potential explanation for the significant improvements observed across all psychological outcomes, independent of treatment allocation, with blinding of treatment assignment remaining throughout.

Furthermore, the trial specifically targeted individuals experiencing stress or anxiety, who may be actively motivated to improve their wellbeing and particularly responsive to expectancy-driven effects. Whilst some evidence suggests certain subgroups may be particularly sensitive to expectancy effects (43), this has not yet been investigated in this demographic group, this is an interesting avenue for future research.

Similarly, the Hawthorne effect offers a related explanation, where individuals modify or improve their behaviour because of knowing it is being assessed or observed (44). Which may have led participants to consciously or unconsciously report improvements, irrespective of treatment allocation, this is likely heightened in self-report psychological measures. Finally, the structured nature of trial participation, including repeated self-monitoring of psychological well-being, may itself have conferred meaningful psychological benefit. The introduction of a supplement-taking routine, increased self-awareness of well-being may have produced therapeutic effects independent of treatment allocation. Evidence suggests that daily self-monitoring may increase feelings of empowerment in depressed out-patients (45). These findings raise important methodological considerations for nutritional intervention trials, more broadly. Notably, should expectancy effects be viewed as confounding variables to be controlled and minimised, or are they a potential mechanism to exert beneficial effects irrespective of intervention? The lack of reporting in this area highlights the need for greater consensus in how they should be interpreted and considered in nutritional intervention study design.

Crucially, the mushroom blend produced significant improvements over and above these participatory effects on the primary outcome, with trend-level evidence for secondary outcomes, which strengthens confidence in the psychological benefits. Nonetheless, the difficulty in fully disentangling treatment-specific effects from participatory benefits underscores the importance of placebo-controlled designs and highlights the need for greater transparency in how these effects are reported and interpreted in RCTs. Whether the magnitude of participatory effects observed here is specific to this demographic or intervention, or is reflective of the broader field, certainly warrants further investigation.

The trial has contributed valuable information to the rapidly growing body of literature on functional mushrooms. This double-blind, placebo-controlled, three-arm randomised controlled study design, demonstrated that 4 weeks of supplementation with a Lion’s Mane and Reishi blend may reduce anxiety in Gen Z women reporting elevated, but subclinical symptoms. Exploratory evidence indicated similar benefits for stress, however given the trend-level of the secondary findings, these results should be interpreted with caution. The intervention was well-tolerated, but whether the observed differences translate into meaningful, or clinically significant benefits for young women with subclinical stress or anxiety has not yet been established. Determining this is a clear priority for future research. As results are limited to this specific population, it is also recommended that future studies repeat this work in more diverse populations.

Recruitment challenges meant the intended sample size was not achieved (N = 84 of an intended *N* = 135), which raises concerns about statistical power and the potential risk of Type II error, particularly for outcomes where treatment effects may be more subtle. Notably, a significant treatment effect was observed for the primary outcome, with post-hoc comparisons indicating moderate-to-large effect sizes; secondary outcomes showed similar patterns at trend-level following FDR correction. However, confidence intervals around these estimates were wide, reflecting the reduced sample size and indicating that the precise magnitude of these effects remains uncertain. Therefore, replication in adequately powered trials is necessary before firm conclusions can be made, and the possibility that more subtle treatment effects were undetected also cannot be excluded. A further limitation is the absence of mechanistic or biomarker data, which prevents determining the biological pathways which may underpin the effects on anxiety and stress outcomes. This represents a significant gap, not only within the present study, but also across the functional mushroom field. Future trials should incorporate biomarker measurement alongside cognitive and mood measures to begin establishing mechanistic pathways.

Additionally, key methodological questions remain unanswered, including optimal dosage and intervention duration, bioavailability of active compounds, supplement composition, and the duration of potential benefits. Addressing these methodological limitations should be a priority for the field, without this information meaningful comparisons across published studies remain difficult, and the true efficacy of mushroom-based products cannot be fully established.

Notwithstanding these limitations, the incorporation of a preliminary sort-and-rank study ensured intervention outcomes aligned with participant priorities, further strengthening the relevance of these findings to the target population and offers a model for participant-informed outcome selection in future nutritional-intervention trials.

## 5 Conclusion

This trial provides evidence that a mushroom blend containing Lion’s Mane and Reishi improved the primary anxiety outcome (GAD-7), relative to Lion’s mane alone in Gen-Z women. Exploratory, trend-level findings on secondary stress and anxiety measures suggest a similar beneficial pattern, but they did not survive correction for multiple comparisons and should be interpreted cautiously. A secondary *post hoc* comparison also tentatively indicated lower anxiety scores in the blend condition 2 weeks after cessation of supplementation, providing preliminary indication of some longevity effects that warrants dedicated investigation in future trials, ideally with dose-matched single-ingredient and combination arms to isolate the contribution of Reishi too. Interestingly, significant improvements across all psychological outcomes were observed irrespective of treatment condition, suggesting that trial participation itself resulted in wellbeing benefit. This highlights the importance of placebo-controlled designs in nutritional intervention research and the need for greater transparency in reporting and discussing participatory effects in the literature, particularly when reporting improvements within active treatment groups, without showing efficacy over placebo. Overall, these findings emphasise the complexity of designing nutritional intervention trials with subjective mood outcomes. While the mushroom blend shows some promise, future research should prioritise disentangling the contributions of individual mushrooms and the synergistic relationship of combination blends. Crucially, key methodological questions including optimal dosage, blend composition, bioavailability and intervention duration must be thoroughly investigated to advance the functional mushroom field.

## Data Availability

The raw data supporting the conclusions of this article will be made available by the authors, without undue reservation.
