# Supplementary material for: The effects of four-week supplementation of Lion’s mane and a Lion’s mane and Reishi blend on prioritised mood and wellbeing measures in young women
Source: Front Nutr. 2026 Oct 20;13:1887408. doi: 10.3389/fnut.2026.1887408 (PMC13727649; doi:10.3389/fnut.2026.1887408)
Supplement: Supplementary file 1 [file Table_1.DOCX]

Supplementary Material

# 1 Sort and Rank Methodology and Results

A sort and rank trial was conducted to determine which areas of poor mental well-being were most important to Gen Z women who self-identified as stressed and/or anxious. Data collection occurred between January and February 2024. Ethical approval was obtained from the Northumbria University Ethics Online System (Reference 5917).

## 1.1 Participants

Thirty-eight women participated in the trial, all were classified as Gen Z and born between 1997 and 2006 (mean age = 19.39, SD = 1.71). Participants self-reported as feeling stressed and/or anxious, without a pre-existing diagnosis of a psychiatric disorder. Sample characteristics and suitability were confirmed by completion of the Perceived Stress Scale (PSS) and Generalised Anxiety Disorder (GAD-7) questionnaire, which confirmed participants were on average moderately stressed (mean score = 23.45) and exhibiting mild clinical anxiety (mean score = 9.29).

## 1.2 Materials and Procedure

Fifty-four statements were developed based on DSM-5 diagnostic criteria for acute stress disorder, generalised anxiety disorder, depressive disorders and ADHD and validated questionnaires assessing these disorders, due to the high prevalence and co-morbidity of these conditions within the target population. The full list of statements can be found in Supplementary Materials.

The sort and rank task was completed online using EQ Web Sort (Banasick, 2025), participants initially sorted the statements into three broad categories: “Most important”, “No strong feelings” and “Least important”. They then completed a structured ranking task, placing each statement on a forced quasi-normal distribution grid ranging from -6 (least important) to +6 (most important). Each level of the ranking grid contained a fixed number of slots, ensuring a consistent distribution across participants.

## 1.3 Data Analysis

Data from the sort and rank task were analysed descriptively, with statements assigned a score from -6 (least important) to +6 (most important) based on its position in the participants forced distribution grid. Mean rank scores were calculated to examine overall trends in perceived importance. Cumulative percentages were calculated to further interpret the relative importance of each statement. This represented the proportion of participants who assigned each statement a rank of less than +2, which corresponds to the top 16 ranked items on the 13-point scale. With a lower cumulative percentage indicating that a greater percentage of participants rated the statement as highly important.

*Results*

Participants consistently prioritised symptoms related to anxiety, fatigue and stress, presented in Table 1.

**Table 1** Top-ranked symptoms of poor mental well-being in Gen Z women

| **Rank** | **Statement** | **% Scored > +2** | **Mean Rank** |
| --- | --- | --- | --- |
| 1 | Excessive anxiety and worry | 59.5 | 2.89 |
| 2 | Feeling mentally tired/exhausted | 56.8 | 2.64 |
| 3 | Feeling physically tired/exhausted | 51.4 | 1.67 |
| 4 | Feeling close to panic | 43.2 | 1.27 |
| 5 | Overwhelming feelings of stress | 43.2 | 2.45 |
| 6 | Feelings of worthlessness | 43.2 | 2.08 |
| 7 | Low self esteem | 43.2 | 1.91 |
| 8 | Lack of motivation | 40.5 | 1.94 |
| 9 | Difficulty working up the initiative/motivation to do things | 40.5 | 2.00 |
| 10 | Unable to muster any energy | 35.1 | 1.16 |

Note: “% scored > +2” indicates the percentage of participants who assigned the statement a rank of greater than +2 on the -6 to +6 scale (i.e., who placed the item in their top 16 most important statements). Higher percentages reflect higher perceived importance.

# 2 Sort and Rank Trial - List of statements

1. Inability to experience positive emotions
2. An altered sense of reality
3. ‘Zoning out’ for long periods of time
4. Difficulty falling asleep
5. Difficulty staying asleep
6. Restless sleep
7. Oversleeping
8. Irritable behaviour
9. Angry outbursts
10. Overreacting to situations
11. Inability to wind down
12. Feeling close to panic
13. Experiencing breathing difficulty (in the absence of physical exertion)
14. Problems with concentration
15. Feeling overwhelmed
16. Lack of motivation
17. Lack of organisation
18. Overwhelming feelings of stress
19. Excessive anxiety and worry
20. Feeling restless
21. Fatigue
22. Unable to muster any energy
23. Mind going blank
24. Muscle tension
25. Low mood
26. Diminished interest in socialising
27. Diminished interest in activities/hobbies
28. Low libido
29. Poor appetite
30. Overeating
31. Feelings of worthlessness
32. Feelings of guilt
33. Low self esteem
34. Inability to finish university work/duties in the workplace
35. Avoidance of tasks that require mental effort
36. Being forgetful in daily activities
37. Feeling unable to control important things in your life
38. Feeling nervous
39. Lacking confidence in your ability to handle personal problems
40. Inability to relax
41. Feeling tense or wound up
42. Finding little enjoyment in things you used to enjoy
43. Unable to see the positive/funny side of things
44. Feeling slowed down
45. Unrefreshing sleep
46. Trouble getting out of bed
47. Procrastinating or putting things off to another day
48. Difficulty making decisions
49. Unsatisfied with daily accomplishments
50. Difficulty working up the initiative/motivation to do things
51. Nervous energy
52. Feeling physically tired/exhausted
53. Feeling mentally tired/exhausted
54. Lack of energy for the day

# 3 Intervention Trial - Inclusion and exclusion criteria

## 3.1 Inclusion Criteria

- Identify as a woman
- Are aged 18 to 26 years at the time of giving consent
- Rate themselves as stressed and/or anxious
- Are a native speaker of English or fluent in English

## 3.2 Exclusion Criteria

- Have any pre-existing medical condition/illness which will impact taking part in the study. NOTE: the explicit exceptions to this are controlled hayfever, asthma, hypo/hyperthyroidism, high blood pressure, high cholesterol, reflux, dyslexia/dyscalculia, ADHD, autism.
- Currently taking prescription medications NOTE: the explicit exceptions to this were contraceptive treatments, and those taken ‘as needed’ in the treatment of asthma and hay fever.
- Has relevant food allergies/ intolerances/ sensitivities
- Excessive caffeine intake (> 500 mg per day)
- Had taken dietary supplements e.g. Vitamins, omega 3 fish oils etc. in the last 4 weeks NOTE – Vitamin D and iron supplements were allowed for this trial if they have been advised by GP to increase levels to a normal range and have been taken for at least 4 weeks consistently and were taken consistently throughout the trial.
- Were pregnant, seeking to become pregnant or lactating
- Had taken antibiotics within the past 4 weeks
- Were currently participating in other clinical or nutrition intervention studies, or had done so in the past 4 weeks
- Had been diagnosed with/ undergoing treatment for alcohol or drug abuse in the last 12 months
- Had been diagnosed with/ undergoing treatment for a psychiatric disorder in the last 12 months (this includes medically diagnosed anxiety and depression)
- Suffer from frequent migraines that require medication (more than or equal to 1 per month)
- Had any sleep disorders or take sleep aid medication
- Had any known active infections

# 4 Intervention Trial – Participant Characteristics

**Table 2**

*Participant characteristics at enrolment for continuous variables for ITT sample. N =84*

| **Measure** | **Mean** | **SD** |
| --- | --- | --- |
| Age (years) | 21.93 | 2.49 |
| Education (years) | 16.26 | 2.07 |
| Fruit & veg consumption (portions/day) | 3.22 | 1.46 |
| Caffeine consumption (mg/day) | 106.88 | 92.17 |
| Systolic BP (mm/Hg) | 112.66 | 9.73 |
| Diastolic BP (mm/Hg) | 74.83 | 8.13 |
| Heart Rate (beats per minute) | 76.50 | 11.76 |
| BMI (kg/m^2^) | 23.35 | 4.93 |
| Waist to hip ratio | 0.79 | 0.06 |

**Table 3**

*Participant characteristics at enrolment for categorical variables for ITT population. N = 84*

| **Measure** | **Count** | **%** |
| --- | --- | --- |
| Ethnicity: |  |  |
| White English, Welsh, Scottish, Northern Irish or British  Asian Indian  Asian Chinese  Asian Pakistani | 58 | 69.0 |
|  | 7 | 8.3 |
|  | 3 | 3.6 |
|  | 1 | 1.2 |
| Asian Bangladeshi | 1 | 1.2 |
| Any other Asian background | 3 | 3.6 |
| Black African | 1 | 1.2 |
| Mixed White and Asian | 2 | 2.4 |
| Mixed White and Black African | 1 | 1.2 |
| Mixed – Other mixed background | 1 | 1.2 |
| Other - Arab | 1 | 1.2 |
| Any other White background | 5 | 6.0 |
| Wear glasses (yes/no) | 31/53 | 37/63 |
| Handedness (left/right) | 10/74 | 12/88 |
| Dietary habits: |  |  |
| Omnivore  Vegetarian  Pescatarian | 66 | 78.6 |
|  | 13 | 15.5 |
|  | 2 | 2.4 |
| Vegan | 1 | 1.2 |
| Other (religious diet, Halal) | 2 | 2.4 |
| Highest level of education: |  |  |
| Level 2 (NVQ Level 2, GCSE A*-C, Young Apprenticeships, BTEC ½ ) | 2 | 2.4 |
| Level 3 (NVQ Level 3, AS & A-Levels, BTEC 3) | 44 | 52.4 |
| Level 4 (NVQ Level 4, Cert of Higher Ed, BTEC 4) | 4 | 4.8 |
| Level 5 (NVQ Level 5, HND, HNC, Dip HE/FE, Found. Deg, BTEC 5) | 3 | 3.6 |
| Level 6 (Bachelor’s Degree, GradDip, Grad Cert, BTEC 6) | 25 | 29.8 |
| Level 7 (Masters, PGDip, PG Cert, BTEC 7) | 6 | 7.1 |

**Table 4**

*Medical History Summary*

| **Medical Condition** | **Count** |
| --- | --- |
| ADHD | 2 |
| Allergy to penicillin | 2 |
| Allergy to animals | 1 |
| Anaemia | 1 |
| Asthma | 1 |
| Historic depression | 2 |
| Dust allergy | 1 |
| Dyslexia | 1 |
| Eczema | 2 |
| Suspected endometriosis | 2 |
| Eosinophilic esophagitis | 1 |
| Hayfever | 4 |
| Hidradenitis suppurativa | 1 |
| Historic anxiety | 1 |
| Hypothyroidism | 1 |
| Iron deficiency | 1 |
| Suspected irritable bowel syndrome | 1 |
| Migraines | 2 |
| Psoriasis | 1 |
| Raynauds | 1 |
| Suspected Red S Syndrome | 1 |
| Scoliosis | 2 |
| Sesame seed allergy | 1 |
| Travel sickness | 1 |

**Table 5**

*Medication Use Summary. Regular medication uses by 39 participants*

| **Medication** | **Count** |
| --- | --- |
| Contraception | 25 |
| Antihistamines | 5 |
| Iron supplements | 5 |
| Other Supplements/vitamins (all stopped at enrolment, 4-week washout prior to randomisation) | 5 |
| Vitamin D | 3 |
| Period pain relief | 2 |
| Thyroxine | 1 |
| Inhaler | 1 |
| Creams for skin conditions | 2 |
| Travel sickness prevention | 1 |

# 5 Intervention Trial – Mood Questionnaires

## 5.1 Generalised Anxiety Disorder Assessment (GAD-7) (Williams, 2014)

A seven item, self-report anxiety questionnaire that assess status over the previous 2 weeks. The outcome of the questionnaire is reported as a score between 0 and 21, with 0 indicating no anxiety and 21 indicating the highest level of anxiety. Scores are clinically categorised as: 0-4 (none), 5-9 (mild), 10-14 (moderate) and 15-21 (severe).

## 5.2 Hospital Anxiety and Depression Scale (HADS) (Snaith & Zigmund, 1986)

A 14-item scale measuring the severity of anxiety and depressive symptoms, over the last two weeks. The outcome of the anxiety subscale is reported as a score between 0 and 21. With scores divided into four stages: 0-7 (Normal), 8-10 (Mild), 11-15 (Moderate), 16-21 (Severe).

## 5.3 Perceived Stress Scale (PSS) (Cohen et al., 1983)

10-item scale which uses responses ranging from ‘never’ to ‘very often’ to provide a measure of perceived stress over the previous month. Individual scores on the PSS can range from 0 to 40, with higher scores indicating higher perceived stress. Scores 0-13 are considered low stress; 14-26 considered moderate stress; 27-40 considered high stress.

## 5.4 Stress Visual Analogue Scales (S-VAS)

Participants complete 4 visual analogue scales assessing how anxious, stressed, relaxed and calm they feel (Not at all (0) - Extremely (100)). An average 'stress' score is created using this data, this score ranges from 0 to 100, with a higher score indicating higher perceived stress.

## 5.5 Chalder Fatigue Scale (Chalder et al.,1993)

This 14-item scale measures the symptoms of mental and physical fatigue. Using Likert style scoring, individuals score in a range from 0 to 33. With a higher score indicating higher levels of fatigue.

## 5.6 Rosenberg’s Self Esteem Scale (Rosenberg, 1965)

A 10-item measure of global perception of self-esteem. An individual will score from 0 to 30. With a lower score indicating lower self-esteem.

**Table 6.**

**Chronic performance on the mood questionnaire measures at all timepoints for ITT population.**

Baseline scores (plus standard error) were collected on Day 1. Data presented includes mean and SE for all assessments. Final three columns showing test statistics (F), associated probabilities (*p*) and adjusted q values for secondary outcomes (negative affect).

| **Outcome** | **Treatment group** | **N** | **Day 1** | | **Day 7** | | **Day 14** | | **Day 21** | | **Day 28** | | **Day 42** | | **Effect** | **F** | ***p*** |  |
| --- | --- | --- | --- | --- | --- | --- | --- | --- | --- | --- | --- | --- | --- | --- | --- | --- | --- | --- |
|  |  |  | **Mean** | **SE** | **Mean** | **SE** | **Mean** | **SE** | **Mean** | **SE** | **Mean** | **SE** | **Mean** | **SE** |  |  |  | Adjusted q value |
| GAD-7  (score range 0 -21) | Placebo | 24 | 9.72 | .48 | - | - | - | - | - | - | 5.59 | .51 | 6.77 | .62 | **Treatment**  **Day**  **Treatment*Day** | **3.99**  **84.97**  **2.68** | **.022**  **<.001**  **.034** | **-**  **-**  **-** |
|  | MB | 25 | 10.36 | .49 | - | - | - | - | - | - | 4.16 | .51 | 4.95 | .56 |  |  |  |  |
|  | LMM | 28 | 10.15 | .47 | - | - | - | - | - | - | 6.50 | .47 | 6.64 | .54 |  |  |  |  |
| HADS (score range 0 -21) | Placebo | 24 | 11.03 | .48 | 9.25 | .51 | 8.56 | .52 | 7.43 | .54 | 6.82 | .52 | 9.07 | .59 | Treatment  **Day**  **Treatment*Day** | 2.03  **26.29**  **2.12** | .136  **<.001**  **.023** | .404  **<.001**  .072 |
|  | MB | 25 | 11.49 | .49 | 9.66 | .52 | 8.49 | .54 | 7.23 | .52 | 6.04 | .51 | 6.18 | .56 |  |  |  |  |
|  | LMM | 28 | 11.35 | .47 | 9.90 | .48 | 8.56 | .48 | 8.71 | .49 | 8.10 | .48 | 7.83 | .55 |  |  |  |  |
| Chalder (score range 0 -33) | Placebo | 24 | 17.14 | .64 | - | - | - | - | - | - | 10.44 | .68 | - | - | Treatment  **Day**  Treatment*Day | .38  **141.10**  .14 | .684  **<.001**  .866 | .747  **<.001**  .866 |
|  | MB | 25 | 17.86 | .65 | - | - | - | - | - | - | 10.72 | .68 | - | - |  |  |  |  |
|  | LMM | 28 | 17.44 | .62 | - | - | - | - | - | - | 11.04 | .63 | - | - |  |  |  |  |
| Self Esteem (score range 0 -30) | Placebo | 24 | 15.74 | .43 | - | - | - | - | - | - | 18.08 | .46 | - | - | Treatment  **Day**  Treatment*Day | .93  **68.47**  .85 | .401  **<.001**  .430 | -  -  - |
|  | MB | 25 | 15.36 | .44 | - | - | - | - | - | - | 18.44 | .46 | - | - |  |  |  |  |
|  | LMM | 28 | 15.68 | .42 | - | - | - | - | - | - | 19.14 | .42 | - | - |  |  |  |  |
| PSS (score range 0 -40) | Placebo | 24 | 23.67 | .69 | - | - | - | - | - | - | 19.79 | .73 | - | - | Treatment  **Day**  **Treatment*Day** | 1.64  **85.33**  **3.45** | .202  **<.001**  **.036** | .404  **<.001**  .072 |
|  | MB | 25 | 24.48 | .70 | - | - | - | - | - | - | 16.86 | .73 | - | - |  |  |  |  |
|  | LMM | 28 | 24.27 | .67 | - | - | - | - | - | - | 19.35 | .68 | - | - |  |  |  |  |
| Stress VAS (score range 0 -100) | Placebo | 24 | 54.47 | 2.77 | 50.27 | 2.93 | 47.67 | 3.00 | 44.52 | 3.10 | 39.78 | 2.97 | 52.68 | 3.42 | Treatment  **Day**  Treatment*Day | .29  **18.65**  1.39 | .747  **<.001**  .182 | .747  **<.001**  .243 |
|  | MB | 25 | 60.34 | 2.83 | 53.58 | 3.01 | 51.96 | 3.11 | 45.73 | 3.00 | 34.38 | 2.93 | 41.63 | 3.20 |  |  |  |  |
|  | LMM | 28 | 59.48 | 2.69 | 49.29 | 2.77 | 47.56 | 2.77 | 44.65 | 2.81 | 37.05 | 2.74 | 40.46 | 3.15 |  |  |  |  |

**Table 7.**

**Chronic performance on the mood questionnaire measures at all timepoints for PP population.**

Baseline scores (plus standard error) were collected on Day 1. Data presented includes mean and SE for all assessments. Final two columns showing test statistics (F) and associated probabilities (*p*).

| **Outcome** | **Treatment group** | **N** | **Day 1** | | **Day 7** | | **Day 14** | | **Day 21** | | **Day 28** | | **Day 42** | | **Effect** | **F** | ***p*** |  |
| --- | --- | --- | --- | --- | --- | --- | --- | --- | --- | --- | --- | --- | --- | --- | --- | --- | --- | --- |
|  |  |  | **Mean** | **SE** | **Mean** | **SE** | **Mean** | **SE** | **Mean** | **SE** | **Mean** | **SE** | **Mean** | **SE** |  |  |  | Adjusted q value |
| GAD-7 (score range 0 -21) | Placebo | 22 | 9.78 | .50 | - | - | - | - | - | - | 5.57 | .59 | 5.85 | .74 | **Treatment**  **Day**  Treatment*Day | **3.68**  **75.15**  1.58 | **.029**  **<.001**  .184 | **-**  **-**  **-** |
|  | MB | 23 | 10.41 | .51 | - | - | - | - | - | - | 4.55 | .58 | 4.70 | .71 |  |  |  |  |
|  | LMM | 27 | 10.25 | .47 | - | - | - | - | - | - | 6.54 | .48 | 6.68 | .57 |  |  |  |  |
| HADS (score range 0 -21) | Placebo | 22 | 11.09 | .52 | 8.80 | .58 | 8.39 | .58 | 7.15 | .65 | 6.97 | .60 | 8.53 | .71 | Treatment  **Day**  Treatment*Day | 1.88  **24.06**  1.53 | .157  **<.001**  .128 | .314  **<.001**  .256 |
|  | MB | 23 | 11.60 | .53 | 9.87 | .58 | 8.52 | .62 | 7.32 | .60 | 5.76 | .59 | 6.47 | .72 |  |  |  |  |
|  | LMM | 27 | 11.44 | .49 | 10.06 | .52 | 8.73 | .51 | 8.73 | .53 | 8.16 | .50 | 7.75 | .59 |  |  |  |  |
| Chalder (score range 0 -33) | Placebo | 22 | 17.22 | .67 | - | - | - | - | - | - | 10.69 | .80 | - | - | Treatment  **Day**  Treatment*Day | .26  **123.87**  .05 | .776  **<.001**  .953 | .774  **<.001**  .956 |
|  | MB | 23 | 17.94 | .68 | - | - | - | - | - | - | 10.96 | .77 | - | - |  |  |  |  |
|  | LMM | 27 | 17.63 | .64 | - | - | - | - | - | - | 10.97 | .65 | - | - |  |  |  |  |
| Self Esteem (score range 0 -30) | Placebo | 22 | 15.63 | .38 | - | - | - | - | - | - | 17.76 | .46 | - | - | Treatment  **Day**  **Treatment*Day** | 1.46  **98.37**  **3.19** | .240  **<.001**  **.047** | -  -  - |
|  | MB | 23 | 15.27 | .39 | - | - | - | - | - | - | 19.50 | .44 | - | - |  |  |  |  |
|  | LMM | 27 | 15.48 | .36 | - | - | - | - | - | - | 18.97 | .37 | - | - |  |  |  |  |
| PSS (score range 0 -40) | Placebo | 22 | 23.62 | .71 | - | - | - | - | - | - | 20.01 | .85 | - | - | **Treatment**  **Day**  **Treatment*Day** | **3.37**  **80.56**  **5.13** | **.040**  **<.001**  **.008** | .160  **<.001**  **.032** |
|  | MB | 23 | 24.52 | .73 | - | - | - | - | - | - | 15.91 | .82 | - | - |  |  |  |  |
|  | LMM | 27 | 24.42 | .68 | - | - | - | - | - | - | 19.46 | .69 | - | - |  |  |  |  |
| Stress VAS (score range 0 -100) | Placebo | 22 | 54.17 | 2.89 | 49.20 | 3.26 | 46.65 | 3.30 | 44.80 | 3.71 | 38.98 | 3.40 | 47.15 | 4.05 | Treatment  **Day**  Treatment*Day | .49  **15.43**  .57 | .613  **<.001**  .842 | .774  **<.001**  .956 |
|  | MB | 23 | 60.46 | 2.97 | 53.69 | 3.25 | 52.11 | 3.53 | 46.80 | 3.36 | 35.46 | 3.31 | 44.57 | 4.06 |  |  |  |  |
|  | LMM | 27 | 59.36 | 2.76 | 49.90 | 2.93 | 47.90 | 2.86 | 44.12 | 2.98 | 36.34 | 2.80 | 40.38 | 3.32 |  |  |  |  |

# 6 Safety Analysis Summary

**Table 8**

*Results of Fisher’s Exact Tests for Adverse Events determined to be potentially related to treatment consumption*

| **Adverse Event** | **Treatment Group** | **Reported (n)** | **Not reported (n)** | ***p*-value** | **FDR –adjusted *p-*value** |
| --- | --- | --- | --- | --- | --- |
| Bloating | Placebo | 1 | 28 | 1.00 | 1.00 |
|  | MB | 0 | 27 |  |  |
|  | LMM | 0 | 29 |  |  |
| Chest pain | Placebo | 0 | 28 | 1.00 | 1.00 |
|  | MB | 0 | 27 |  |  |
|  | LMM | 1 | 29 |  |  |
| Diarrhoea | Placebo | 0 | 28 | .66 | 1.00 |
|  | MB | 1 | 27 |  |  |
|  | LMM | 0 | 29 |  |  |
| Dizziness | Placebo | 0 | 28 | 1.00 | 1.00 |
|  | MB | 0 | 27 |  |  |
|  | LMM | 1 | 29 |  |  |
| Headache | Placebo | 10 | 28 | .43 | 1.00 |
|  | MB | 4 | 27 |  |  |
|  | LMM | 8 | 29 |  |  |
| Heartburn | Placebo | 1 | 28 | .76 | 1.00 |
|  | MB | 1 | 27 |  |  |
|  | LMM | 0 | 29 |  |  |
| Lightheaded | Placebo | 1 | 28 | 1.00 | 1.00 |
|  | MB | 0 | 27 |  |  |
|  | LMM | 0 | 29 |  |  |
| Migraine | Placebo | 1 | 28 | 1.00 | 1.00 |
|  | MB | 0 | 27 |  |  |
|  | LMM | 1 | 29 |  |  |
| More stress and anxiety when treatment taken late | Placebo | 0 | 28 | 1.00 | 1.00 |
|  | MB | 0 | 27 |  |  |
|  | LMM | 1 | 29 |  |  |
| Nausea | Placebo | 1 | 28 | 1.00 | 1.00 |
|  | MB | 1 | 27 |  |  |
|  | LMM | 2 | 29 |  |  |
| Stomach pain | Placebo | 2 | 28 | .76 | 1.00 |
|  | MB | 0 | 27 |  |  |
|  | LMM | 1 | 29 |  |  |

# 7 Supplementary Materials References

Chalder, T., Berelowitz, G., Pawlikowska, T., Watts, L., Wessely, S., Wright, D., & Wallace, E. P. (1993). Development of a fatigue scale. *Journal of psychosomatic research*, *37*(2), 147-153.

Cohen S, Kamarck T, Mermelstein R. (1983). A global measure of perceived stress. J Health Soc Behav 24:385–96

Rosenberg, M. (1965). Rosenberg self-esteem scale. *Journal of Religion and Health*.

Snaith, R. P., & Zigmond, A. S. (1986). The hospital anxiety and depression scale. *British medical journal (Clinical research ed.)*, *292*(6516), 344.

Williams, N. (2014). The GAD-7 questionnaire. *Occupational medicine*, *64*(3), 224-224.
